# Behavioural variation among workers promotes feed-forward loops in a simulated insect colony

**DOI:** 10.1098/rsos.220120

**Published:** 2022-03-02

**Authors:** Carrie Easter, Ellouise Leadbeater, Matthew J. Hasenjager

**Affiliations:** ^1^ School of Biology, University of Leeds, Leeds LS2 9JT, UK; ^2^ Department of Biological Sciences, Royal Holloway, University of London, Egham TW20 0EX, UK

**Keywords:** network motif, social insects, feed-forward loop, agent-based model, personality, behavioural syndrome

## Abstract

Coordinated responses in eusocial insect colonies arise from worker interaction networks that enable collective processing of ecologically relevant information. Previous studies have detected a structural motif in these networks known as the feed-forward loop, which functions to process information in other biological regulatory networks (e.g. transcriptional networks). However, the processes that generate feed-forward loops among workers and the consequences for information flow within the colony remain largely unexplored. We constructed an agent-based model to investigate how individual variation in activity and movement shaped the production of feed-forward loops in a simulated insect colony. We hypothesized that individual variation along these axes would generate feed-forward loops by driving variation in interaction frequency among workers. We found that among-individual variation in activity drove over-representation of feed-forward loops in the interaction networks by determining the directionality of interactions. However, despite previous work linking feed-forward loops with efficient information transfer, activity variation did not promote faster or more efficient information flow, thus providing no support for the hypothesis that feed-forward loops reflect selection for enhanced collective functioning. Conversely, individual variation in movement trajectory, despite playing no role in generating feed-forward loops, promoted fast and efficient information flow by linking together otherwise unconnected regions of the nest.

## 1. Introduction

In many group-living species, social interaction patterns play an important role in shaping fitness outcomes, such as by impacting access to social information, the likelihood of cooperation or exposure to pathogens [1]. Beyond an individual's direct connections, evolutionary fitness may further be influenced by the patterning of interactions at the group level. For instance, a minority of highly interactive individuals can accelerate the spread of information or disease throughout a population by linking together otherwise unconnected individuals [2] and modular social structures can contain the spread of information within tightly knit communities [3]. These group-level properties are probably especially important in eusocial insect colonies in which only one or a few colony members reproduce, such that the fitness of individual workers is tightly linked to colony collective performance [4].

In the absence of any central control, interactions among workers regulate task allocation through a distributed process, ensuring that the effort devoted to various tasks matches a colony's internal needs and external conditions [5,6]. Meeting the demands of this regulatory role is likely to favour different interaction patterns to those that are observed in social systems where individual success is key. In other words, a social insect worker's position in the network is often less important for its fitness than higher level network properties [5–7]. For instance, among-individual variation in interaction frequency in harvester ants (*Pogonomyrmex barbatus*) generates networks with a few highly interconnected individuals while the majority of workers remain only weakly connected [2]. Networks structured in this way permit rapid information transfer throughout a population and can thereby facilitate swift collective responses to changing conditions [2].

**Figure 1.**
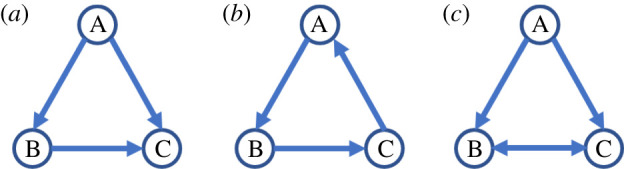
Examples of triangle configurations. (*a*) A transitive triangle, or feed-forward loop, in which one individual, A, has two outgoing edges and another, C, has two incoming edges. (*b*) A cyclic triangle, in which all individuals have one outgoing and incoming edge. (*c*) A triangle with a bidirectional edge connecting B and C.

Social network analysis has emerged as a key approach for quantifying variation in social connectivity and investigating its ecological and evolutionary consequences [1,8,9]. A useful means to gain insight into a network's functionality is to deconstruct it into its constituent subcomponents [10]. A network can, for instance, be described in terms of the different three-node subgraphs (or triads) from which it is composed. Because subgraphs differ in their functional properties [11–13], over-representation of a given subgraph within a network (relative to its typical representation within an ensemble of appropriately randomized networks) can suggest the processes or functions that have helped to shape that network. For example, food webs display an over-representation of simple chains derived from trophic interactions—e.g. species A consumes species B, which in turn consumes C [10]—whereas gene transcription networks contain an over-abundance of a triadic configuration known as the ‘feed-forward loop’ (figure 1*a*; [12]), whereby a gene A transcriptionally regulates the activity of a second gene B, and both A and B jointly regulate a third gene C. As feed-forward loops are well-suited to carry out signal processing tasks (e.g. amplifying responses to external environmental cues), this structural feature may reflect the regulatory function of these networks [11].

The regulatory role of interaction networks within social insect colonies may likewise be reflected in their constituent subcomponents. In common with other biological regulatory systems, the antennation patterns of harvester ants (*P. californicus*), which play a key role in transmitting task-relevant information between colony members, show an over-representation of feed-forward loops [7], at least relative to their appearance in size- and density-matched random graphs. Similarly, dominance relationships in the eusocial wasp *Ropalidia marginata* are made up predominately of feed-forward loops and are involved in regulating worker activity through agonistic interactions [14]. However, the way in which such network structures develop in social insect colonies is unclear. In contrast with other biological regulatory networks, where the relationships between nodes are relatively stable (e.g. one gene produces a transcription factor that activates or inhibits another gene), the nodes in social insect interaction networks represent individual workers that engage in brief pairwise interactions with one another and often lack stable relationships. The patterning of interactions among workers instead arises from the behaviour of individuals that influence their likelihood of interacting. In some cases, the presence of feed-forward loops might simply reflect the tendency of a particular type of relationship to be transitive—e.g. in the dominance networks of *R. marginata* [14], if worker A is dominant over B and B is dominant over C, A is likely to also be dominant over C. Feed-forward loops consequently tend to form in the network. Yet even in this case, the formation of dominance relationships is dependent on other aspects of behaviour that influence individuals' likelihood of interacting, such as their spatial location on the nest. If A and C never interact, a feed-forward loop will never form between A, B and C. Furthermore, the reason for the development of feed-forward loops in interaction networks that lack such hierarchical organization (e.g. the antennation patterns of *P. californicus*) is less clear and suggests that more subtle behavioural mechanisms may be responsible for generating this structural feature in these populations.

The structure of a feed-forward loop inherently implies among-individual variation in contact patterns, as each node differs in the number of incoming and outgoing connections (or edges). Insect workers express substantial among-individual variation along a number of behavioural axes that may contribute to the generation of such network structures [15]. For instance, workers vary in the proportion of time that they are actively engaged in tasks: e.g. a minority of workers often carry out the majority of work [16–20], with some workers even appearing to specialize in inactivity [21]. Workers also vary in their spatial behaviour within the nest. This can partly be determined by activity levels—more active individuals will tend to cover more ground per unit time—but can also result from variation in movement patterns. Some *P. barbatus* workers, for example, walk very sinuous paths, causing them to occupy relatively restricted regions within the nest, while others walk straighter paths and so roam more extensively [2]. Such among-individual variation in activity and space-use may play central roles in shaping social contact patterns by influencing the likelihood that particular individuals will contact one another. For example, workers that move in straighter paths will probably contact a greater number of nest-mates than workers that remain restricted to small regions within the nest. That different pairs of individuals vary in their likelihood of interacting further suggests that random graph models, which typically assume an equal probability of interaction between any pair of nodes, may not be the most appropriate null model with which to assess the presence of network motifs in empirical social insect interaction networks.

Here, we construct an agent-based model to investigate how among-individual variation in activity and movement patterns in a simulated insect colony contributes to the formation of interaction networks dominated by feed-forward loops. We further consider how this variation drives the speed and efficiency of information flow within the colony. Our model is not designed to reproduce the dynamics of any specific species. Rather, we seek to evaluate structural and functional consequences of patterns of behavioural variation that are commonly observed across eusocial insects [15,22], with a particular focus on how such variation shapes patterns of physical contact between workers (e.g. antennation), which are central in regulating collective behaviour [2,6,7]. We first predict that, by determining how frequently individuals contact others and how diverse those contacts are, among-individual variation in activity and movement will drive over-representation of feed-forward loops in the resulting interaction networks. We further predict that, when these sources of variation are treated as a ‘behavioural syndrome’ (i.e. individual activity and patterns of movement covary, such that the most active agents also walk straighter paths), they will have a synergistic effect on the production of feed-forward loops by emphasizing among-individual variation in space-use. Second, due to the tendency of feed-forward loops to move information in a directional manner [11,12], we predict that patterns of behavioural variation that generate feed-forward loops will also lead to faster and more efficient information flow, in the sense that fewer interactions will be needed to drive the spread of information throughout a colony [7].

## 2. Methods

### 2.1. Agent-based model

**Figure 2.**
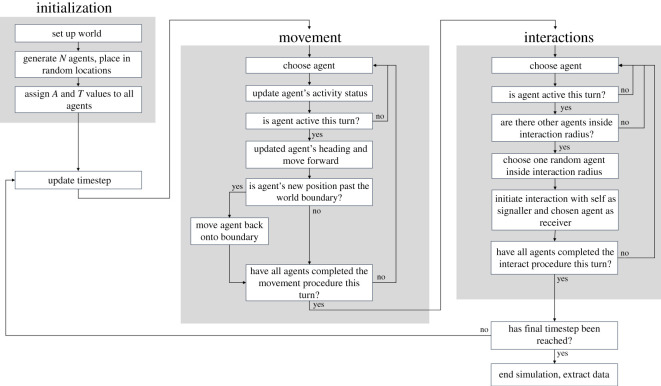
Flowchart describing the main model processes, including model initialization, agent movement, agent interactions, time-step updates and data extraction.

Our model was created in the agent-based modelling platform NetLogo (v. 5.2.0; https://ccl.northwestern.edu/netlogo/) and is available in the electronic supplementary material. We briefly describe the model's main processes below (also summarized in figure 2). The full model description, which follows the ODD (overview, design concepts, details) protocol [23], can be found in the electronic supplementary material.

#### 2.1.1. Model initialization

The model consists of a 50 × 50 grid of square ‘patches’ (the ‘nest’) containing the agent population (*N_default_* = 100), which represent workers within a social insect colony. At the beginning of each simulation, each agent (*i*) is placed in a random location and assigned its activity level (*A_i_*) and turning index (*T_i_*). The value of *A_i_* determines how mobile an agent is within the nest and, under certain conditions, its likelihood of transmitting information to others upon physical contact. Conceptually, active agents represent individuals currently engaged in some task within the nest—e.g. nest construction, food distribution, recruitment—with the possibility of transmitting task-relevant information to individuals that they come into contact with. The value of *T_i_* determines how sinuous an agent's walking path is—more sinuous paths result in greater spatial fidelity as individuals move away from their starting position more slowly. Agent movement occurs during discrete time steps (*t_default_* = 5000 steps) and the order in which agents act is selected randomly on each step. Once all agents have had the opportunity to move, agents can initiate interactions with nearby nest-mates, with the order of action once more randomly determined. See ‘Agent movement’ and ‘Agent interactions’ below for an explanation of these processes.

Activity levels and turning indices can be either uniform or variable across the population and are centred around the population means, *A_m_* and *T_m_*, respectively. When activity levels are uniform, all agents are assigned the same value, *A_i_ = A_m_*. When activity levels are variable, values are randomly drawn from an exponential distribution with mean *A_m_,* such that most agents are relatively inactive, while a few are highly active. Similar distributions of activity have been observed across multiple social insect species (e.g. ants [24]; stingless bees [20]; bumblebees [19]). When turning indices are uniform, all agents are assigned the same value, *T_i_ = T_m_*. When turning indices are variable, values are first randomly drawn from an exponential distribution with mean T*_m_*. These values are subsequently modified (see full ODD model in the electronic supplementary material) such that most agents have relatively high turning indices, resulting in more tortuous movement paths, whereas a few agents have low turning indices, and so move in straighter paths (as observed in [2]). In addition, when both activity levels and turning indices are variable, values of *A* and *T* can either be uncorrelated or negatively correlated across agents. If uncorrelated, these values are assigned to agents independently of each other. If negatively correlated, these values are paired such that the agent with the highest value of *A* also has the lowest value of *T*, and so on, thus generating a population where more active agents also tend to move in straighter paths. This was done in order to explore whether such a behavioural syndrome [15] may especially contribute to the formation of feed-forward loops, given that active individuals with a greater potential to initiate contact (see Agent interactions, below) would also potentially contact a greater diversity of individuals, due to reduced spatial fidelity.

#### 2.1.2. Agent movement

**Figure 3.**
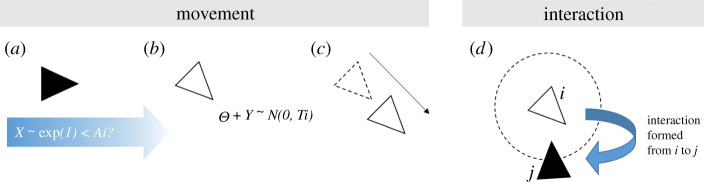
Movement and interaction processes are performed by all agents each time step. During the movement process, (*a*) an agent, *i*, begins the time step as inactive (black shading). If a value, *X*, drawn from an exponential distribution with a mean of 1 is less than *i*'s activity level, *A_i_*, (*b*) the agent becomes active (white shading) and updates its heading, then (*c*) moves forward. During the interaction process, (*d*) any agent that is currently active (white) forms a directed interaction to a randomly selected agent within its interaction radius (dashed circle). In this example, active agent *i* forms a directed interaction to inactive agent *j*.

Agents move in a correlated random walk [25] governed by their activity level and turning index (figure 3*a–c*). Agent *i* will only move during time step *t* if it is ‘currently active’. An agent is considered to be active on a given time step if $\propto <A_{i}$, where ∝ is randomly drawn from an exponential distribution with mean 1. Accordingly, if *A_m_* is set to 1, half of the agents in the population will, on average, be active on any given time step, comparable to observed activity patterns in several social insect species [17,18,26]. If agent *i* is active during time step *t*, its current heading is updated as $\theta_{i,t+1}=\theta_{i,t}+\delta \theta$, where δθ represents the change in direction drawn from a normal distribution with mean 0 and standard deviation $T_{i}$ (cf. [27,28]). A higher value of $T_{i}$ will therefore result in a more tortuous movement path and consequently greater spatial fidelity within the nest. After updating its heading, agent *i* then moves forward by one body length (equivalent to 0.5 patches). Agents are prevented from moving past the nest boundaries to capture the physical constraints present within a social insect nest. If forward movement would cause *i* to move past a boundary, its x and/or y coordinate is set to that of the boundary to ensure that it does not move past.

#### 2.1.3. Agent interactions

Agents can form pairwise, directed interactions with one another upon coming into contact (figure 3*d*). The formation and direction of interactions are both determined by agent activity by default (but see Experiment ii, where these constraints are relaxed). Each agent has an ‘interaction radius’ of 0.5 patches (equivalent to one body length) which determines when they are in physical contact with each other. An interaction radius equal to one body length was used because worker interactions often involve physical contact (e.g. antennation and food sharing) and observations on multiple species suggest one body length is well within the range at which workers can detect nest-mates [29,30]. If agent *i* is currently active and has at least one other agent within its interaction radius at time step *t*, it forms an outgoing connection to one random agent, *j*, within its interaction radius. Agent *i* is therefore considered the information signaller and *j* the information receiver. Our assumption that information transfer is linked to activity in this way reflects a situation often seen in social insect species, where knowledgeable individuals actively transmit information to others, e.g. the honeybee waggle dance [31]. In other circumstances, greater activity can be positively related to the likelihood of acquiring information from nest-mates—e.g. when an ant detects the cuticular hydrocarbons or food-associated odours borne by nest-mates that it encounters [6]. We therefore investigated an alternative ‘to-active’ condition, where information tends to flow towards more active individuals, which produced qualitatively similar results (electronic supplementary material, figures S1 and S2).

### 2.2. Data collection

For every interaction that occurred throughout the simulations, we logged the following information: the ID of both agents involved, the interaction's direction and the time step during which the interaction occurred. This information was exported as a .csv file at the end of each simulation.

### 2.3. The experiments

Across several experiments, we investigated how the production of feed-forward loops within an interaction network is influenced by individual variation in activity and movement patterns, and the influence this has on within-colony information flow. Below, we describe the scenarios tested in each experiment and the statistical analyses of the data. In all cases, we used a population size of 100 agents and ran simulations for 5000 time steps. All statistical analyses were carried out in R v. 4.0.3 [32]. Generalized least squares (GLS) models were fit using maximum-likelihood estimation in the *nlme* package [33]. A GLS framework allowed us to incorporate variance structures that modelled observed heterogeneity in residual spread where applicable [34]. Model selection was performed on the basis of Akaike's information criterion corrected for sample size (AICc) [35]. When a single model was strongly supported by the data (Akaike weight greater than or equal to 0.95), inferences were based on that model. Otherwise, model-averaging was used across the minimal set of models whose summed Akaike weights were greater than or equal to 0.95. Model selection was carried out using the *MuMIn* package [36].

#### 2.3.1. Effects of behavioural variation on triangle transitivity

##### 2.3.1.1. Model parameters and scenarios tested

By determining how agents move, among-individual variation in activity and movement pattern is expected to influence how frequently (and with whom) individuals interact, thereby shaping the structure of the resulting interaction networks. We therefore compared simulations in which among-individual variation was present for activity level, turning index or both to simulations in which these traits remained uniform across the population (table 1). We ran 100 simulations for each of the following conditions: (i) uniform, where *A_i_* and *T_i_* were set to *A_m_* and *T_m_*, respectively, across all agents; (ii) activity variable, where *A_i_* (but not *T_i_*) varied across agents; (iii) TI variable, where *T_i_* (but not *A_i_*) varied across agents; (iv) uncorrelated, where both traits varied within a population, but activity variation was independent of variation in turning index and (v) correlated, where both traits varied within a population and *A_i_* was negatively correlated with *T_i_*. For each condition, interaction initiation was determined by agent activity and during interactions, active agents generated outgoing edges.

**Table 1.** Behavioural conditions.

condition	activity level, *A_i_*	turning index, *T_i_*	behavioural syndrome?
uniform	uniform	uniform	NA
activity variable	varies	uniform	NA
TI variable	uniform	varies	NA
uncorrelated	varies	varies	$A_{i}$ and $T_{i}$ are independent
correlated	varies	varies	$A_{i}$ and $T_{i}$ are negatively correlated

For all conditions, $A_{m}=1$ and $T_{m}=60$. Setting $A_{m}$ to 1 means that, on average, 50% of agents will be active on a given turn. Similar inactivity levels are commonly observed across social insect species [17,18,26]. A mean turning index of 60 is comparable to that observed in multiple ant species [2,37,38]. In addition, a sensitivity analysis showed that our results were robust to a range of values for $A_{m}$ and $T_{m}$ (electronic supplementary material, tables S1–S5 and AS5).

##### 2.3.1.2. Statistical analysis

To evaluate the role of among-individual variation in generating feed-forward loops, we compared mean triangle transitivity, *t*_tri_, across the different conditions [13]. Triangle transitivity quantifies the tendency of triangles (i.e. triadic configurations in which all three dyads are connected) to be transitive (i.e. form a feed-forward loop) rather than cyclic (figure 1*a,b*). This value is scaled relative to the expected proportion of transitive triangles, such that a value of 0 indicates that the proportion of transitive triangles does not differ from random expectations and a value of 1 indicates that all triangles are transitive (figure 1*a*) and none are cyclic (figure 1*b*).

We first extracted weighted time-aggregated networks from the interaction lists collected after each simulation (see Data collection) [39]. Time-aggregated networks were then converted into binary directed networks as follows: non-interacting dyads received a value of 0; dyads in which all interactions were in a single direction were linked by a binary edge with that same directionality; and for dyads in which interactions occurred in both directions, directionality of the binary edge corresponded to whichever direction greater than 50% of interactions occurred in. If an equal number of interactions occurred in both directions, that dyad was linked by a bidirectional binary edge (figure 1*c*).

For each simulation run, time-aggregated networks were built over increasingly larger time windows (starting from *t* = 0) until the resulting binary network contained at least *n* edges, where *n* ranged from 150 to 1500 in increments of 150. For each combination of condition (table 1) and network density (*n* = 150, 300, …, 1500), 10 binary networks were obtained; only one binary network was extracted from a given simulation run. Triangle transitivity was then calculated for each binary network as described in [13] (see the electronic supplementary material for more details on triangle transitivity calculations). Triangle transitivity values were used as the response variable in a GLS model with behavioural condition (table 1), network density and their interaction as predictors. Prior to analysis, network density was standardized by subtracting the mean and dividing by the standard deviation.

To further compare the structure of the time-aggregated networks, triad significance profiles (TSP) for the seven possible triangle configurations were also obtained for each non-uniform behavioural condition. TSPs are vectors of normalized Z-scores that quantify the representation of each triangle configuration relative to that expected from a null model. Here, the uniform condition represented the null model of interest, where all individuals expressed the same mean activity and turning index. Using time-aggregated networks containing 1000 binary edges, we first obtained Z-scores for each triangle configuration in each non-uniform simulation as

$$Z_{i}=\frac{N_{Obs}-\;N_{Uniform}}{s.d._{Uniform}},$$

where $N_{Obs}$ is the frequency of a given triangle configuration in the time-aggregated network and $N_{Uniform}$ and $s.d._{Uniform}$ are, respectively, the mean and standard deviation of the frequency of that triangle configuration across 100 networks from the uniform condition. Z-scores were then normalized as follows:

$$\mathrm{Normalized}\;Z-\mathrm{score}=\;\frac{Z_{i}}{\surd \left(\sum Z_{i}^{2}\right)}.$$

#### 2.3.2. Effects of activity on triangle transitivity

##### 2.3.2.1. Model parameters and scenarios tested

As well as determining an agent's probability of moving on time step *t,* $A_{i}$ also directly influences both the initiation and directionality of interactions. To disentangle the impact of these latter two elements on triangle transitivity, we modified the model to run the following four conditions: (i) activity determines whether interactions are initiated, but not directionality. In this condition, interactions are initiated only by currently active agents as described above, but the interaction's direction is assigned randomly, such that both agents have a 0.5 probability of becoming the information signaller. (ii) Activity determines interaction directionality, but not initiation. In this condition, each agent, regardless of its activity status, has a 0.5 probability of initiating an interaction on each time step, provided that there is at least one agent within its interaction radius. The direction of the interaction is then determined by the relative activity levels of the two agents, such that agents that tend to be more active are more likely to become signallers. Specifically, the probability of an interaction being directed from agent *i* to agent *j* is proportional to $\frac{A_{i}}{A_{i}+A_{j}}$. (iii) Neither interaction initiation nor directionality is determined by activity. In this condition, all agents have a 0.5 probability of initiating an interaction on each time step, with the direction of any resulting interactions determined randomly. (iv) Both interaction initiation and directionality are determined by activity (this is the default condition, as described above). For each condition, we ran 100 simulations each for the activity variable and uniform treatments (table 1). For all conditions tested here, $A_{m}=1$ and $T_{m}=60$.

##### 2.3.2.2. Statistical analysis

Network density had only a weak effect on triangle transitivity (see Results). As such, for each simulation, we extracted a binary network that contained 1000 edges as previously described. Values of $t_{tri}$ were then compared across conditions using GLS models.

#### 2.3.3. Speed and efficiency of information flow

To evaluate how among-individual variation influenced information flow within the population, we simulated a simple diffusion process through 100 simulations for each condition specified in table 1. The first agent to initiate an interaction in a simulation was treated as the initially informed individual. Naive individuals that received an incoming interaction from an informed individual became informed themselves and capable of transmitting that information onwards. As in [40], we estimated transmission speed as the time step at which greater than or equal to 50% of agents were informed in each simulation, T50. To evaluate how efficiently information spread, we also recorded the cumulative number of outgoing interactions from informed individuals (whether to naive or informed agents) that occurred by T50. Our measure thus equates efficiency with maximizing the spread of information while minimizing the number of interactions.

Substantially more interactions occurred during simulations with uniform activity levels across agents than those in which activity levels varied. All else being equal, higher interaction rates should result in a more rapid spread of information [2]. To disentangle the effects of the patterning of interactions from the total number of interactions on the diffusion process, we randomly selected and removed 20% of interactions in each simulation run in which activity levels were uniform across agents, prior to simulating the diffusion process. This resulted in a similar interaction rate across all runs without systematically altering the patterning of interactions generated by different behavioural conditions. GLS models were used to compare T50 and the efficiency of information flow across behavioural conditions. Response variables were log-transformed to meet assumptions of normality.

## 3. Results

### 3.1. Effects of behavioural variation on triangle transitivity

**Figure 4.**
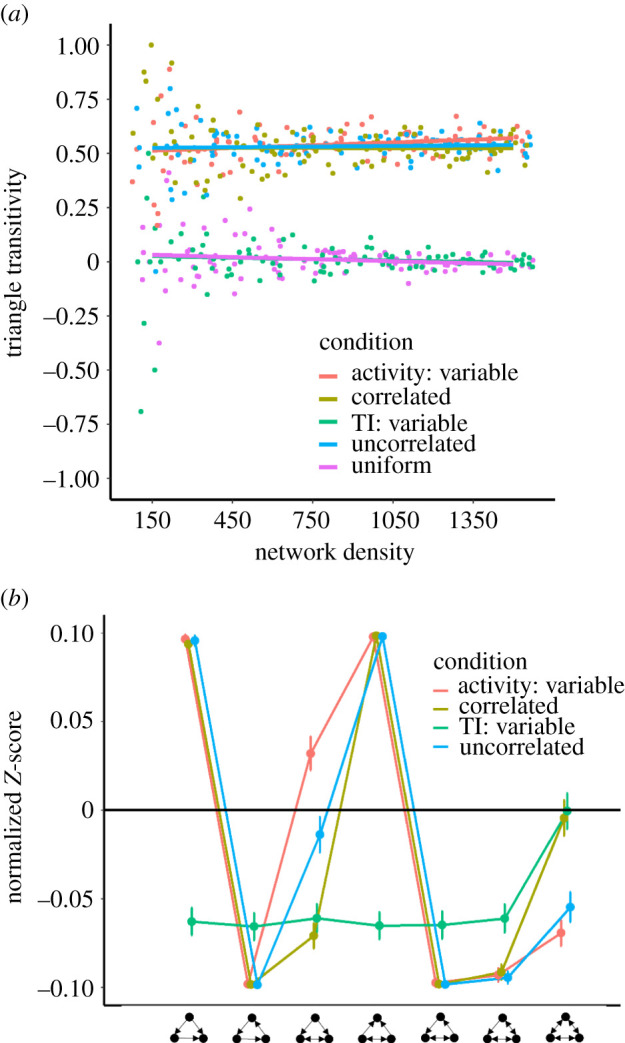
(*a*) Triangle transitivity as a function of behavioural condition and network density. Active agents generated outgoing edges. Lines depict predicted values. (*b*) TSP comparing the relative significance of triangle configurations across the non-uniform behavioural conditions, relative to the uniform condition. Normalized Z-scores were averaged across 100 simulation runs for each condition; bars indicate the standard errors. For both graphs, points are horizontally jittered to improve clarity.

There was strong evidence for an effect of behavioural condition on triangle transitivity, and the triadic configurations of the social network as a whole (figure 4). Consistent across a range of network densities, networks were dominated by transitive (rather than cyclic) triangles (indicated by relatively greater *t*_tri_) when activity levels varied among individuals, whereas when all individuals were equally active, the number of transitive triangles matched random expectations—i.e. *t*_tri_ ≈ 0 (figure 4; table 2). As binary networks became more dense (i.e. more connections), variation in *t*_tri_ decreased across simulations, though there was little change in mean *t*_tri_ (figure 4*a*).

**Table 2.** GLS model of triangle transitivity as a function of behavioural condition. Model-averaged estimates (MAE) and unconditional standard errors (USE) derived from the two best-supported models given in the electronic supplementary material, table S1 ($\sum w_{i}=0.999$). Intercept taken as condition = uniform. A variance structure was incorporated that allowed for heterogeneous residual spread across network density, dependent on condition. *N* = 1000 simulations.

parameter	MAE	USE	95% CI
intercept	0.007	0.010	−0.012, 0.026
condition = activity variable	0.538	0.016	0.506, 0.571
condition = TI variable	0.002	0.011	−0.020, 0.023
condition = uncorrelated	0.526	0.013	0.501, 0.552
condition = correlated	0.518	0.012	0.494, 0.542
network density	−0.010	0.008	−0.027, 0.006
activity variable * network density	0.024	0.016	−0.008, 0.056
TI variable * network density	0.003	0.009	−0.015, 0.020
uncorrelated * network density	0.013	0.012	−0.009, 0.036
correlated * network density	0.010	0.010	−0.011, 0.030

When individuals varied in only their turning indices, the abundance of fully connected triads on the whole were reduced compared with the uniform condition (figure 4*b*). Among-individual variation in turning index also altered spatial structuring within the nest. Individuals with more sinuous walking paths (i.e. high *T_i_*) tended to cluster into localized areas, while those with straighter walking paths navigated a greater proportion of the nest (electronic supplementary material, figure AS3).

### 3.2. Effects of activity on triangle transitivity

**Figure 5.**
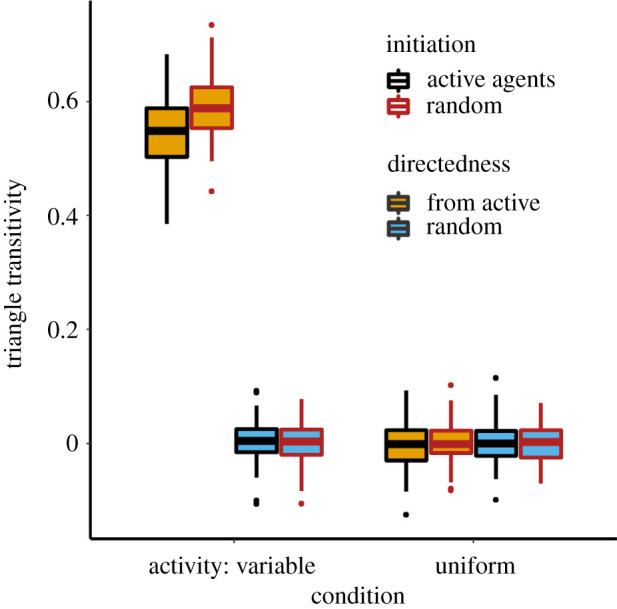
Boxplots depicting the effects of activity on triangle transitivity. Thick lines indicate medians, while the boxes indicate the interquartile range. Whiskers extend to 1.5x the interquartile range. Condition refers to whether activity levels varied or were uniform among agents. Interactions were either initiated randomly (red outlines) or by a currently active agent moving within the interaction radius of another agent (black outlines). The direction of an initiated interaction was either assigned randomly (blue fill) or according to an agent's current activity (yellow fill).

Triangle transitivity varied according to a three-way interaction between condition (uniform versus activity variable) and activity-based influences on interaction initiation and directionality (figure 5; table 3). Triangle transitivity was significantly higher when activity levels varied among individuals and activity determined interaction direction (mean *t_tri_* = 0.569) compared with instances in which these conditions were not met (mean *t_tri_* = 0.001; figure 5) (see electronic supplementary material, figure S4, for TSP). Put simply, over-representation of feed-forward loops emerged when some individuals were more likely than others to generate directed network connections.

**Table 3.** Parameter estimates from GLS model of triangle transitivity as a function of behavioural condition, interaction initiation and interaction directionality. Estimates derived from best-supported model ($\sum w_{i}=0.999$). Intercept taken as condition = uniform with both initiation and direction of interactions determined by activity. A variance structure was incorporated that allowed for heterogeneous residual spread across both condition and directional variants. *N* = 800 simulations.

parameter	estimate	s.e.	95% CI
intercept	−0.001	0.004	−0.008, 0.007
condition = activity variable	0.547	0.006	0.534, 0.559
random initiation	−0.0004	0.006	−0.012, 0.011
random direction	0.003	0.005	−0.007, 0.013
activity variable * random initiation	0.046	0.009	0.028, 0.064
activity variable * random direction	−0.546	0.008	−0.562, −0.530
random initiation * random direction	−0.001	0.007	−0.015, 0.013
activity variable * random initiation * random direction	−0.045	0.012	−0.068, −0.023

### 3.3. Speed and efficiency of information flow

**Figure 6.**
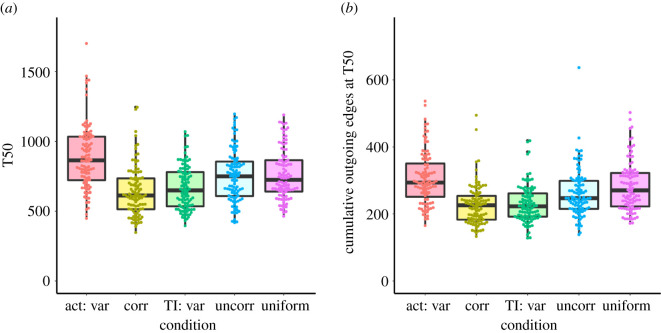
Speed and efficiency of information flow under different behavioural conditions. Boxplots show (*a*) T50, the time step at which greater than or equal to 50% of individuals were informed, and (*b*) the cumulative number of outgoing interactions from informed individuals until T50. Lower values, respectively, correspond to faster and more efficient transmission. Thick lines indicate medians, while the boxes indicate the interquartile range. Whiskers extend to 1.5x the interquartile range.

The speed of information flow—measured by T50, the time step at which 50% of agents were informed—varied across conditions (figure 6*a*; table 4). T50 was highest (i.e. information spread most slowly) when individuals varied only in activity level. When individuals independently expressed variation in both activity and turning index, the speed of information flow was similar to when no variation was present in either trait. T50 was lowest (i.e. information spread most rapidly) either when individuals varied in turning index alone or when this variation was negatively correlated with variation in activity levels.

**Table 4.** Parameter estimates from linear model of T50 (log-transformed) as a function of behavioural condition. Estimates derived from best-supported model ($\sum w_{i}>0.999$). Intercept taken as condition = uniform. *N* = 500 simulations.

parameter	estimate	s.e.	95% CI
intercept	6.611	0.025	6.562, 6.659
condition = activity variable	0.148	0.035	0.080, 0.217
condition = TI variable	−0.136	0.035	−0.204, −0.068
condition = uncorrelated	−0.018	0.035	−0.086, 0.051
condition = correlated	−0.179	0.035	−0.248, −0.111

Efficiency of information flow, measured by the number of outgoing interactions from informed agents that had occurred until T50 was reached, also varied across conditions (figure 6*b*; table 5). Information spread most efficiently (fewest outgoing connections) either when individuals varied only in turning index or when variation in activity level and turning index were negatively correlated with one another. When mean activity and turning index were uncorrelated, transmission efficiency was similar to the condition in which no individual variation was present. Information transmission was least efficient (most outgoing connections) when individuals varied in mean activity level alone.

**Table 5.** Parameter estimates from linear model of transmission efficiency (log-transformed) as a function of behavioural condition. Estimates derived from best-supported model ($\sum w_{i}>0.999$). Intercept taken as condition = uniform. *N* = 500 simulations.

parameter	estimate	s.e.	95% CI
intercept	5.612	0.025	5.563, 5.660
condition = activity variable	0.081	0.035	0.012, 0.149
condition = TI variable	−0.201	0.035	−0.270, −0.133
condition = uncorrelated	−0.080	0.035	−0.148, −0.011
condition = correlated	−0.216	0.035	−0.284, −0.148

## 4. Discussion

The superorganismal nature of eusocial insect colonies means that natural selection is increasingly expected to act on colony-level traits [4], such as the ability to generate robust, yet flexible, colony-level responses to ecological challenges. Collective coordination relies on interactions that transfer information between nest-mates, raising the possibility that natural selection has acted on the behavioural algorithms that determine whether and how workers interact. Using a simple agent-based model, we demonstrate that among-individual variation in the likelihood of sending outgoing (or receiving incoming) links is sufficient to generate an over-abundance of a triadic network substructure known as the ‘feed-forward loop’ (figure 1*a*). This motif is commonly found in biological regulatory networks where it performs various signal processing tasks, e.g. discriminating persistent signals from short-lived pulses [10,11], and is also over-represented within social insect interaction networks, where similar regulatory roles have been demonstrated [7,14]. Nevertheless, our model found that feed-forward loops alone had little impact on information transmission processes. Rather, among-individual variation in movement patterns (either alone or as part of a behavioural syndrome) promoted faster and more efficient information transfer, despite contributing little to the production of feed-forward loops. Our model thus demonstrates how collective properties that support colony functioning can be tuned by modifying both the behavioural variation present among workers and correlations across traits.

Insect workers often vary considerably in their activity levels [16–19], with a minority of individuals generally carrying out most of the work [19,20,24]. These individuals can also play a key role in transmitting task-relevant information through interactions with nest-mates [15]. Honeybee (*Apis mellifera*) foragers, for example, vary dramatically in their likelihood to produce recruitment dances, even when collecting from identical resources [41]. Similarly, highly active ‘keystone individuals' catalyse worker activity in ant colonies [42]. We therefore linked activity in our model to the likelihood of directed information transfer between individuals and found that, when this criterion was satisfied, individual variation in activity drove the production of feed-forward loops within the interaction networks. However, given that other effects of activity variation in our model (e.g. total distance moved) were unimportant for the generation of these motifs, it seems likely that any behavioural trait that (i) varies among individuals and (ii) directly influences the directionality of pairwise interactions (e.g. the direction of information transfer) could drive an over-abundance of feed-forward loops.

One such trait may be the propensity to interact with nest-mates. For example, honeybees vary in their likelihood to engage in trophallactic food-sharing interactions [43], with some individuals potentially specializing in offering food [44]. Dominance interactions are also characterized by clear directional relationships—indeed, transitive relationships are a common feature in dominance hierarchies, in both insects [14] and other taxa [13]. In various ant species, for example, trophallaxis is generally directed from subordinate to dominant individuals [22]. Variation in knowledge or past experience is also likely to promote transitive network structures when it results in directed information transfer among workers. For example, more experienced *Temnothorax albipennis* ants are more likely to engage in tandem runs, where they directly lead naive followers to a resource [45]. Similarly, it has been suggested that in the grass-cutting ant (*Acromyrmex heyeri*), workers initially sacrifice foraging efficiency in order to more rapidly provide nest-mates with information about newly discovered foraging resources [46].

Nevertheless, while transitive network structures are a potentially common feature of social insect colonies, whether they offer any functional benefit remains unclear. Previous analyses of empirical social insect networks have shown that an over-representation of feed-forward loops could reflect selection for more efficient information transfer in insect colonies [7,14]. However, our model found that the speed and efficiency of information transfer was unrelated to the proportion of transitive triangles in the population social network. For example, among-individual variation in activity alone produced comparable triangle transitivity levels compared with when individuals varied in both activity and turning index, but the former was associated with relatively slow and inefficient transmission compared with the latter. This suggests that the effects of feed-forward loops on collective functioning are likely to be context-dependent.

It is also possible that feed-forward loops confer regulatory properties beyond those considered here. For instance, in transcriptional networks, feed-forward loops can dampen responses to external signals to ensure that transient signals are ignored [11]. A similar role may be present in insect colonies by limiting collective responses to weak signals about low-quality resources and thereby promote effective worker allocation. Workers often vary in their response thresholds to task-related stimuli, with some requiring relatively little stimulation to begin work, while others must experience substantially higher intensities of task-related stimuli before acting [22]. Feed-forward loops may regulate worker activation by limiting responses to weak task-relevant stimuli, while ensuring sufficient stimulation (e.g. multiple signals from active workers) is received by inactive workers when more help is truly needed. It is also possible that, in some cases, the production of feed-forward loops is simply an inadvertent by-product of the behavioural variation present within insect colonies and not itself a target of selection. Previous work has shown, for example, that the frequency and nature of lower level dyadic interactions play a key role in determining the types of triadic configurations that can arise in a network [47].

It is worth noting that our model assumed that behavioural variation remained constant over time. In reality, an individual's activity and/or propensity to interact with others may shift in response to factors such as worker loss, changes in colony food stores or the discovery of a new resource, and these changes may in turn influence how information is transferred through the colony [19,29]. Nevertheless, while our model represents a simplified transmission scenario, it demonstrates clearly how variation in simple individual-level behaviours can significantly impact colony-level information transfer. It also highlights the challenge in inferring the functionality of dynamic systems from knowledge of the static network structure alone. Within insect colonies, interactions are often brief and stable relationships between particular individuals are generally absent. Under such conditions, very different patterns of interaction can give rise to similar network structures when aggregated over time [39]. Whereas previous analyses of the function of feed-forward loops have focused on systems with relatively fixed relationships (e.g. gene regulatory networks; [11]), within insect colonies, the timing and order of interactions is of critical importance. Indeed, when we simulated information flow on the static networks derived from our time-ordered interaction lists, rather than on the time-ordered interactions themselves, we found that in agreement with previous studies [11,14] information spread more efficiently on networks characterized by an over-representation of transitive triangles (electronic supplementary material, tables S6 and S7 and figure S6).

In contrast with among-individual variation in activity, individual variation in movement paths often improved both the speed and efficiency of information transfer in our model, despite having limited impact on the generation of network transitivity. Spatial behavioural variation was included in our model in terms of walking path sinuosity, causing some individuals to remain in restricted areas of the nest, while others traversed the entire nest space [2,28] (electronic supplementary material, figure S3). Under certain conditions, such variation in space-use allowed for faster and more efficient information transfer through the colony. In particular, these effects were observed either when individuals varied in path sinuosity alone or when activity levels were negatively correlated with turning indices across the population—that is, agents with sinuous walking paths tended to be inactive while those with straighter walking paths were often active. In many eusocial insect species, similar patterns of space-use variation have been observed. Bumblebees (*Bombus terrestris*), for example, perform irregular ‘excited’ runs throughout the nest after returning from successful foraging trips, which serve to increase foraging activity in other workers by rapidly distributing pheromones, and potentially through physical contacts [48,49]. Similarly, red harvester ants (*P. barbatus*) vary in the sinuosity of their walking trajectories, which influences their interaction frequency. Ants with straighter walking paths contact more nest-mates than those with more tortuous paths [2]. Our model is consistent with the hypothesis that such variation in connectivity facilitates rapid information flow throughout the population due to workers with straighter walking paths linking isolated clusters of individuals [2].

It should be noted, however, that the adaptiveness of fast, efficient information transfer is highly context dependent. In response to predation, for example, insect colonies are likely to benefit from rapid alarm propagation that can quickly marshal colony defences [50,51], whereas rapid information transfer may be less valuable in a foraging context. Instead, the *regulation* of information transmission in response to environmental feedback is key to ensuring worker effort is divided according to the quality of resources [52], and colonies that show restraint in foraging efforts can often be more successful [53,54]. In addition, behavioural variation that promotes fast and efficient information transfer may also promote faster transmission of pathogens. In this case, we would expect natural selection to favour collective responses to the infiltration of pathogens that limit unnecessary interactions. On exposure to pathogens, for example, some ant species switch from allogrooming to self-grooming—or even isolate themselves from other workers completely—thus reducing potential infection of healthy nest-mates [55,56]. Similarly, nest architecture can influence disease spread throughout a colony, with physically or behaviourally segmented nests tending to dampen the spread of disease [56,57].

## 5. Concluding remarks and future directions

**Figure 7.**
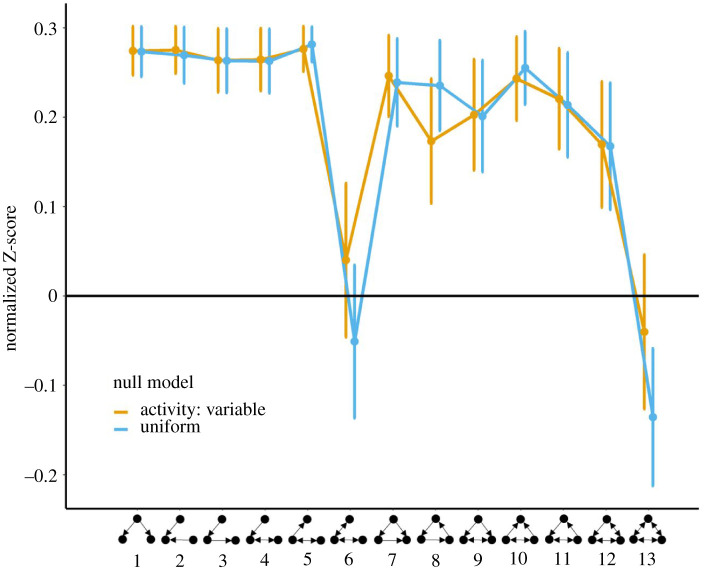
TSP comparing the relative significance (measured by Z scores) of triangle configurations found in Waters and Fewell's [7] data compared with null models generated from our simulation using either activity variable or uniform conditions. Normalized Z-scores were averaged across 12 networks with varied sizes and across 100 simulation runs for each condition; bars indicate the standard errors.

Further research is required to establish whether and how feed-forward loops impact the collective functioning of social insect colonies. Central to these efforts is quantifying the extent to which feed-forward loops and other network motifs are present within colony interaction networks. A common approach is to compare empirical networks with Erdős-Rényi random networks matched for size and density, yet these null models often lack biological and physical relevance [58,59]. For example, random graphs typically assume that all individuals are equally likely to interact, thus ignoring spatial and temporal constraints on interactions (e.g. two individuals that generally occupy opposite sides of the nest are unlikely to interact). A potential application of our model lies in the generation of spatially explicit null models, tuned to a particular system, that will enable realistic comparison with empirical data. To illustrate this point, we reanalysed previously published data on interaction networks of the ant *P. californicus* [7], using our agent-based simulations to generate spatially explicit null models that match the empirical data in network size and density (see electronic supplementary material for details on this analysis). Comparing the empirical networks with random graph models, the original study concluded that feed-forward loops were over-represented, while three-cycles (figure 1*b*) matched expected frequencies (figure 3 in [7]). Conversely, our method suggests that both substructures are over-represented in the empirical data relative to the simulated data (figure 7). We stress that our reanalysis does not invalidate the findings of [7]—indeed, our model is not parametrized appropriately for their data in terms of ant worker activity and movement. Rather, these results emphasize the important role that selecting a null model plays in the interpretation of network analyses. By offering a means to generate spatially explicit null models, we anticipate that our model will prove useful for future investigations into the mechanisms that drive the structure of animal social networks.

## Data Availability

The simulation model, along with all data and code to reproduce our analyses are available from the Dryad Digital Repository, doi:10.5061/dryad.brv15dv8f [60]. All supplementary analyses are available in the electronic supplementary material [61].
